# Supplementary material for: Modular development enables rapid design of media for alternative hosts
Source: Biotechnol Bioeng. 2021 Oct 18;119(1):59–71. doi: 10.1002/bit.27947 (PMC9298315; doi:10.1002/bit.27947)
Supplement: Supplementary file 1 — Supplementary information. [file BIT-119-59-s001.docx]

**Supporting information:**

**Modular media development enables rapid media design for alternative hosts**

**Code:** The Openblend package at: https://github.mit.edu/lovelab/openblend

**S1**. Synthetic cholesterol supplementation alone does not elicit a productivity enhancement comparable to the multicomponent cholesterol supplement

**S2**. Specific productivity and OD600 at harvest for co-feed supplement screen
